# Data of variability and joint variability of global crop yields and their association with climate

**DOI:** 10.1016/j.dib.2019.103745

**Published:** 2019-03-08

**Authors:** Ehsan Najafi, Indrani Pal, Reza Khanbilvardi

**Affiliations:** a Civil Engineering Department, The City College of New York, The City University of New York, New York City, 10031, USA; b NOAA Center for Earth System Sciences and Remote Sensing Technologies (NOAA-CREST), The City College of New York, The City University of New York, New York City, 10031, USA; c Columbia Water Center, Columbia University, New York City, 10025, USA

**Keywords:** Maize, Rice, Sorghum, Soybean, Climate, Joint variability, RPCA

## Abstract

We present the output data of Robust Principal Component Analysis (RPCA) applied to global crop yield variability of maize, rice, sorghum and soybean (MRSS) as presented in the publication “Climate drives variability and joint variability of global crop yields” (Najafi et al., 2019). Global maps of the correlation between all the principal components (PCs) acquired from the low rank matrix (L) of MRSS and Palmer Drought Severity Index (PDSI), air temperature anomalies (ATa) and sea surface temperature anomalies (SSTa) are provided in this article. We present co-varying countries, impacted cropland areas across global countries, and 10 global regions by climate and the association between PCs and multiple atmospheric and oceanic indices. Moreover, the joint dependency between PCs of MRSS yields are presented using two different approaches.

**Table:** Specifications table

Subject area	*Earth and Planetary Sciences, Environmental Science*
More specific subject area	*Atmospheric Science, Global and Planetary Change*
Type of data	*Table, figure*
How data was acquired	*This data is acquired applying Robust Principal Component Analysis (RPCA) on detrended crop yields of maize, rice, sorghum and soybean of global countries with a complete dataset from 1961 to 2013. Spearman correlation is used to find the association between Principal Components (PCs) and local and large scale climatic patterns. The same method is used to identify co-varying countries and joint dependency between PCs.*
Data format	*Analyzed, processed*
Experimental factors	*Annual country-based crops yields, export, import and production are collected from the Food and Agriculture Organization of the United Nations statistical databases. PDSI, ATa, SSTa and multiple monthly atmospheric and ocean time series were obtained from NOAA/OAR/ESRL PSD. These climatic variables are used to study the links between climatic patterns and crop yield variability. Irrigated and rain fed maps for each crop are combined in order to obtain the global spatial coverage of the croplands.*
Experimental features	*Data were analyzed using RPCA and spearman correlation.*
Data source location	*130, 98, 73 and 37 maize, rice, sorghum and soybean producer countries and global oceans, seas and lakes.*
Data accessibility	*The data are available with this article*
Related research article	*E. Najafi, I. Pal, and R. Khanbilvardi, “Climate drives variability and joint variability of global crop yields”, Sci. Total Environ., doi:* *https://doi.org/10.1016/j.scitotenv.2019.01.172,vol**. 662, pp. 361–372, 2019.**[1]**.*

**Table:** 

**Value of the Data** • Global maps of the association between crop yield variability and Palmer Drought Severity Index, air temperature anomalies and sea surface temperature anomalies are valuable since they improve our understanding about the impact of large-scale and local-scale climate on crops productivity. • The presented correlation analysis between PCs and oceanic and atmospheric indices can be informative regarding to the periodic and predictable nature of many large-scale climatic patterns. • Joint variability of global crops and crop producers presented here can be beneficial for crops trade purposes. • This dataset can be used to compare joint variability between countries and the impact of climate on crops that have used different approaches [2], [3], [11] • This dataset is beneficial for policy decision makers, water and energy managers, government and non-government organizations, stakeholders and insurance companies.

## 1 Data

Concurrent and one year-lag correlation between all the PCs of maize, rice, sorghum and soybean (MRSS) acquired from the low rank matrix (L) of Robust Principal Component Analysis (RPCA) and annual average of PDSI, air temperature anomalies (ATa) and sea surface temperature anomalies (SSTa) (in total 412 maps) are provided in the supplementary. In all the maps, significant correlations (95% confidence) over croplands of MRSS are marked with small black dots. The first few PCs explain a large portion of the crop yield variability, and succeeding PCs account for the remaining variability. The variability explained by each PC is shown at the top of each map. The boundary of the countries with large loading values are highlighted in orange (large positive loading values) and blue (large negative loadings values). These countries co-vary similarly/oppositely (same loading sign value countries co-vary similarly and vice versa), and they explain the variance of that PC the most.

**Fig. 1:**
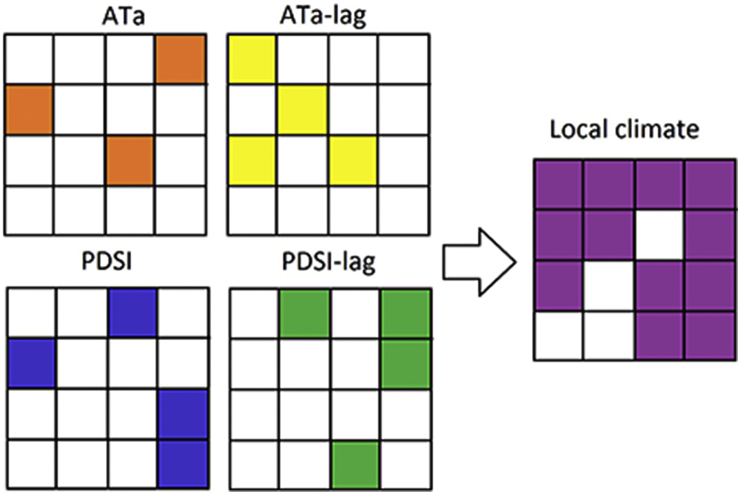
Schematic illustration of overlapping the significant local climate variability over croplands.

Nine tables in the Microsoft Excel Worksheets are included in the supplementary. MRSS producer countries with complete yield dataset from 1961 to 2013 that are used as input for RPCA are presented in Table S1. These countries are categorized in 10 global regions. Table S2 exhibits countries with large positive and negative loading values (co-varying countries). The studied countries are ranked based on MRSS production, export and import in 2013 (Table S3). The area (in hectares) of MRSS croplands in global countries (10 major MRSS producers) that experienced the influence of concurrent and one-year lag of ATa and PDSI are presented in Table S4 (Table S5). In Table S4 and S5 we computed the cropland areas that are impacted by AT or PDSI, in either concurrent or lag phase too (we call it the regions that are impacted by local climate, see Fig. 1). The percentage of the impacted croplands by ATa and PDSI concurrent and lag phases in 10 global regions are exhibited in Table S6. Tables S4, S5 and S6 are based on PC1 to PC3 of the global maps provided in the supplementary. Table S7 demonstrates the concurrent and lag correlation between multiple annual average and seasonal average (December, January, February-DJF) of multiple oceanic and atmospheric indices and PC1 to PC3. Joint dependencies between PC1 to PC3 is presented in Table S8. Table S9, is similar to Table S8, with the difference that PCs are computed based on the low rank matrix of crop yields of 17 countries with complete yield data of all the four crops after 1961.

## 2 Experimental design, materials, and methods

RPCA [4] was applied on detrended crop yields of MRSS. Country based annual crop yield data of these crops from 1961 to 2013 are collected from the Food and Agriculture Organization of the United Nations statistical databases [5]. However, the detrending approach that is used here [6] leads to losing the first and last three years, but each anomaly value captures the information of a 7-year window time span and the final time span is from 1964 to 2010. Yield values of some countries are common in few consecutive years, so this detrending approach leads to undefined values if there are 7 such years with the same consecutive numbers. The same crop yield values over a few years may indicate dataset inaccuracy, hence, in order to improve data reliability these countries are not considered. The final dataset contains 130, 98, 73 and 37 MRSS producer countries (Table S1). Low rank matrix (L) acquired from RPCA is implemented to compute loadings, eigenvalues and scores. Loadings contain uncorrelated PCs [7]. Eigenvalues provide a measure of the variance explained by each PC. RPCA decomposed the L matrix of MRSS into 28, 28, 27 and 20 PCs respectively. Each PC is associated with a score vector. Scores are used to find links between PCs and climatic data set (PDSI, ATa, SSTa, oceanic and atmospheric indices [8]) by means of spearman correlation analysis. In order to obtain the global spatial coverage of MRSS croplands, we combined the irrigated and rain fed maps for each crop [9], [10]. The resulting maps are used to specify the spatial coverage of croplands as well as croplands area (in hectares). We choose co-varying countries (countries with large positive or negative loading values) based on one standard deviation exceedance of loadings values from the mean from both sides in each PC. RPCA applied to global crop yields of MRSS in Argentina, Australia, Brazil, China, Colombia, DR Congo, India, Italy, Mexico, North Korea, Pakistan, Peru, Romania, South Korea, Tanzania, USA and Zimbabwe. These countries produce MRSS and they have complete yield data set from 1961 to 2013. The acquired L matrix is used to compute PCs and identify joint variability between crops (Table S9).
