# Supplementary material for: Data of variability and joint variability of global crop yields and their association with climate
Source: Data Brief. 2019 Mar 8;23:103745. doi: 10.1016/j.dib.2019.103745 (PMC6660635; doi:10.1016/j.dib.2019.103745)
Supplement: Multimedia Component 1 [file mmc1.doc]

**Conflict of interest**

None of the authors has competing/conflicting interests in relation to the issues tackled in this paper.
